# One-Pot Synthesis of Waterborne Polymeric Dispersions Stabilized with Alkali-Soluble Resins

**DOI:** 10.3390/polym10010088

**Published:** 2018-01-18

**Authors:** Massimo Bandiera, Roelof Balk, Maria J. Barandiaran

**Affiliations:** 1 POLYMAT and Departamento de Química Aplicada, Facultad de Ciencias Químicas, University of the Basque Country UPV/EHU, 20018 Donostia-San Sebastián, Spain; massimo.bandiera@ehu.eus; 2 BASF SE, 67056 Ludwigshafen am Rhein, Germany; roelof.balk@basf.com

**Keywords:** emulsion polymerization, electrosteric stabilizer, alkali-soluble resin, grafting, coating

## Abstract

Alkali-soluble resins (ASRs) are a type of electrosteric emulsifiers of high interest because they can profitably improve the features of waterborne dispersions. In this work, they have been synthesized in-situ through a one-pot approach and they have been used as polymeric surfactants for a second emulsion polymerization step in the same reactor. This strategy provides some advantages compared to other polymerization techniques, like the intensification of the process and the absence of organic solvents. Their use can also further reduce the environmental impact of formulations for film-forming applications, since grafting reactions with the particles have been observed and quantified in relation with the synthetic parameters. These chemical linkages with the particles may reduce the leaching and the release of surfactants from polymeric films, for example in water-based coatings or adhesives. The systems have been also studied from the kinetics point of view, finding relevant differences with other electrosterically stabilized processes from the literature, as well as concerns the nucleation mechanism.

## 1. Introduction

Waterborne polymeric dispersions account for about 10% of the world’s polymer production [1], meaning an absolute value of 10 million metric tonnes as dry polymer in 2013, with an increasing trend [2]. Emulsion polymerization is the leading technique to produce such kind of materials, which find application in large volume fields, as adhesives, paper coating, carpet backing and industrial or architectural coatings, as well as biomedical and pharmaceutical applications [2,3]. Hydrophobic colloids are intrinsically unstable systems from the thermodynamic point of view, and in the absence of surfactants, van der Waals forces cause a rapid coagulation of these systems. Apart from stability concerns, the amounts and types of emulsifiers are important for the nucleation process and, in general, for the final properties of a polymeric dispersion produced through emulsion polymerization [1,4]. On the other hand, it is well-assumed that surfactants in emulsion polymerization processes have to be considered as the “necessary evil” [5], since the properties of the resulting formulations, especially for film-forming applications, are seriously worsened. Adhesion, water sensitivity and barrier properties are often compromised because of the migration and/or segregation of molecules of emulsifiers in the film-formation stage [5,6,7,8,9,10]. This induces another important drawback; the migration of the surfactant, especially to the film-air interface, exposes the emulsifier to external conditions. When the latex is for example used as binder for architectural coatings formulations, emulsifiers can leach from roofs or facades and accumulate into the environment. Most of commonly used surfactants have synthetic origin, and have difficulty biodegrading, thus persisting and accumulating in the environment [11]. These problems are particularly relevant when using “conventional” ionic emulsifiers, which act as electrostatic stabilizers. Indeed, the kinetics of migration are basically diffusion-controlled processes, and relatively small molecular weights, as those of many ionic surfactants, imply high mobility in the polymer matrix. From these considerations, nonionic polymeric surfactants represent a valuable alternative to low-molecular weight ionic emulsifiers. Even if strong adsorption on polymeric particles during the polymerization process generally causes inefficient particles nucleation [12], many other properties of the dispersions can be beneficially improved, like the resistance to shear stresses, the sensitivity to electrolytes and the tolerance to freeze–thaw cycles [13,14].

Among the wide possibilities given by polymeric emulsifiers, alkali-soluble resins (ASRs) attracted the interest of researchers because they combine the advantages of polymeric surfactants with other important benefits of the resulting dispersions, like Newtonian-type rheology, good capability of pigments dispersion and improved mechanical features [15,16]. They are random copolymers of mainly (meth) acrylate esters, vinyl aromatics and more hydrophilic monomers like acrylic and methacrylic acid. For usual emulsion polymerization applications, the molecular weights normally range between 5000 Da and 20,000 Da. The presence of anionic charges, above the pK_a_ of the carboxylic acids, allows the dissolution in aqueous media and the possibility of stabilizing colloidal dispersions through electrosteric repulsion forces [17]. Contrary to block copolymers with analogous compositions, that can also stabilize latexes, the implementation in industrial processes is more straightforward, and common free radical polymerization techniques are suitable. Solution polymerization [18], bulk polymerization [19,20] and emulsion polymerization [21,22] can be chosen as alternatives to produce ASRs with different acid values (AVs), molecular weights and compositions. The last strategy, in particular, represents a very interesting option; the synthesis of the resin can be conducted in-situ in a stirred tank reactor through a conventional emulsion (semi) batch process. After a neutralization step, the polymeric particles are dissolved in the aqueous medium and the reactor is already charged with the ASR that can be employed as protective colloid for the stabilization of a second stage polymer. The strategy is versatile and can be applied to many formulations, also with functional monomers, to obtain different molecular weights. The potential use of a single reactor is obviously a feature in the direction of process intensification and, more in general, of Green Chemistry [23]. Besides, it is possible to reduce the use of volatile compounds that may remain in the final dispersion.

Despite of the industrial relevance, there are not many works in open literature dealing with the implementation of in-situ synthesized ASRs as stabilizers on semibatch emulsion polymerization. Most of the studies on ASRs have been performed using commercial resins which are mainly produced in continuous high-temperature processes, being styrene one of the main monomers. With this strategy, it is possible to produce resins with high AV, which are more efficient as stabilizers, since the particles are surrounded by a considerable density of anionic charges. Generally, resins with AVs lower than 110 may have problems of limited solubility in water, whereas values roughly above 300 could cause pronounced water sensitivity of the resulting films [24]. On the contrary, there are only few works describing the synthesis of ASRs by means of emulsion polymerization, and frequently the resin is used as stabilizer after neutralization in batch processes [25].

The objective of this work is therefore to synthesize high-solid content environment-friendly polymeric dispersions stabilized with ASR-type electrosteric stabilizers, using the one-pot approach with fully neutralized ASRs. For this purpose, ASRs with similar low AV but different type of monomers, mainly acrylates or methacrylates, will be synthesized, at two levels of molecular weights, and their aggregation ability analyzed. Moreover, the study aims at shedding light on the kinetic features of these systems, since a reduction of the rate of reaction has been normally observed with electrosteric surfactants and justified with different explanations (formation of stable tertiary radicals after abstraction, diffusional limitation, electrostatic repulsion) [25,26]. Finally, microstructural features including molecular weight and grafting will be estimated and related to the synthetic parameters, in order to provide support to the design of leaching-free waterborne film-forming formulations.

## 2. Experimental

### 2.1. Material

Technical grade monomers methyl methacrylate (MMA, Quimidroga, Barcelona, Spain), ethyl acrylate (EA, Sigma-Aldrich, Steinheim, Germany), styrene (S, Quimidroga), acrylic acid (AA, Sigma-Aldrich), methacrylic acid (MAA, Sigma-Aldrich), butyl acrylate (BA, Quimidroga), butyl methacrylate (BMA, Sigma-Aldrich) were used as received without any further purification, as well as sodium persulfate (NaPS, >99%, Sigma-Aldrich) as initiator. Sodium dodecyl sulfate (SDS, >99%, Sigma-Aldrich) was used to prepare a 15 wt % solution of surfactants in water. 2-Ethylhexyl thioglycolate (EHTG, >95%, Sigma-Aldrich) and 1-butanethiol (99%, Sigma-Aldrich) were used as chain transfer agents (CTAs) to target the desired molecular weights of the resins. Aqueous ammonia 25% was purchased from Merck. Deionized water was used throughout the work.

### 2.2. Emulsion Polymerization Stabilized with In-Situ Synthesised ASR

Two-stage semibatch emulsion polymerization was carried out. In the first stage, ASRs were produced. To study the effect of the composition of the ASR resin, four different formulations were employed as described in Table 1. Two different molar amounts of CTA were used, to target a weight-average molecular weight of about 5000 Da or 10,000 Da. For the second stage, BA or BMA were used as monomers, to point out any eventual difference between acrylates and methacrylates. However, since the two monomers present only moderate differences in terms of water solubility, analogous nucleation behavior is expected.

The reactions were performed in a 1-L jacketed glass reactor equipped with reflux condenser, nitrogen inlet, temperature probe, feeding inlet and stainless-steel agitator rotating at 250 rpm. 

In each reaction, the reactor was charged with the initial charge, and then the temperature was raised to 80 °C under moderate nitrogen flux. After equilibration, an aqueous solution of initiator was added as a shot. After 5 min of agitation, the feeding of the first stage pre-emulsion was started and completed in 40 min. The dispersion was kept under agitation for 10 min of post-polymerization, thereafter ammonia was added as a shot, followed by other 10 min of agitation. Then, the second-stage pre-emulsion was fed over 90 min. The reaction mixture was then kept at 80 °C for additional 120 min before cooling to room temperature.

The ratio resin:monomer was set to 30:70, which is in the range of common industrial applications. The final solids content of the dispersions was 40 wt %. An example of formulation is given in Table 2 to target a resin of 10,000 Da. For the syntheses with 1-butanethiol, EHTG was replaced on molar basis.

### 2.3. Methods

The conversion of monomers was determined gravimetrically. Overall conversions refer to the total weight fed in the reactor at the end of the reaction and the instantaneous conversions are referred to the weight fed at a certain time. 

Z-Average particle diameters were determined by dynamic light scattering (DLS) (ZetaSizer Nano S, Malvern, Malvern, UK) at 25 °C. Before the analysis, the samples taken from the reactor were diluted with deionised water to prevent multiple scattering. The number of particles (*Np*) was calculated from measurements of particle size and the polymer mass in the reactor, assuming full instantaneous conversion, using the following equation:

(1) $$N_{p}=\frac{6w_{p}}{\pi \rho_{p}d_{p}^{3}}$$

where *w_p_* is the mass of the polymer in grams, *ρ_p_* is the density of the polymer particles (g/cm^3^) and *d_p_* is the average particle size obtained from DLS expressed in cm.

The volume average particle size distributions were measured by capillary hydrodynamic fractionation (HDC) with a Matec CHDF3000 equipment. The column was a PL-PSDA Type 2 (Agilent, Santa Clara, CA, USA). Each sample was firstly diluted to 1% solids content, filtered through a 1.2 μm filter and injected with an autosampler (25 μL).

The acid value of the ASRs was calculated from the molar amounts of carboxylic groups in each formulation, assuming as negligible any side reaction decreasing it (e.g., condensation or esterification reactions).

The transmittance of solutions of resin was measured with a Hach DR 6000 spectrophotometer at a wavelength of 600 nm. Measurements were carried out in 1 cm cuvette at 25 °C, background absorption was subtracted with deionized water. Since the measurements were performed after some hours of collection of the sample, it can be assumed that the systems were at equilibrium.

Surface tension measurements were performed at 23 °C and 55% relative humidity using a platinum Du Nouy ring installed on a KSV Sigma 700 tensiometer. The aqueous solutions of ASRs at pH 10 were added through a controlled pump Dosimat 665 onto the water at pH equal to 10 (corrected with ammonia).

Molecular weight distributions and grafting amounts between the ASR and the hydrophobic polymer were quantified by gel permeation chromatography (GPC). Samples were dried until constant weight and diluted in THF (HPLC grade, Scharlau) to a concentration of 2 mg/mL. To 5 mL of the mixture, 35 μL of (trimethylsilyl) diazomethane (solution approximately 2M in diethylether, Acros Organics, Geel, Belgium) were added to methylate the carboxylic acid groups. The samples were let reacting, then the solutions were filtered through a 0.45 μm nylon filter before injection. The GPC equipment consisted of a pump (LC-20A, Shimadzu, Kyoto, Japan), an autosampler (Waters 717), a refractive index detector (Waters 2410) and three columns in series (Styragel HR2, HR4 and HR6 with pore sizes ranging from 102 to 106 Å). Chromatograms were obtained at 35 °C using a THF flow rate of 1 mL/min. The equipment was calibrated using narrow polystyrene standards and the reported values are referred to this calibration. For the determination of grafting, quantifications based on signal intensity of the unmodified low-molecular weight peak attributed to the free resin were performed on the raw chromatograms, as described previously [27]. Reported values can be assumed with an estimated error of 10%.

## 3. Results and Discussion

### 3.1. In-Situ Synthesis of ASRs

All the ASRs were obtained as stable latexes with full conversion, in both the target average molecular weights of the investigation (10 kDa and 5 kDa). The average sizes of the particles were rather similar, between 70 nm and 90 nm, since the nucleation was basically controlled by the amount of surfactant used. After neutralization, ASRs were characterized by optical measurements [21]. It is assumed that clear neutralized resins (i.e., high transmittance) are more homogeneous in composition and more suitable for final applications. Small samples from the reaction mixture were therefore withdrawn after the neutralization step, quickly cooled to room temperature and the transmittance at 600 nm measured. Results are given in Table 3 as average of two measurements and error is given as half of the range.

The results showed that the optical properties were very different, passing from visually almost clear to completely turbid dispersions. High transmittances were observed in resins based on MAA and of bigger size (denoted as 10 kDa, left column on Table 3). This may indicate that the hydrophilic groups are uniformly distributed along the polymeric chains and the addition of ammonia allows a good solubilization of the chains. Interestingly, the resins with the same composition but smaller molecular weight (5 kDa, right column of Table 3) showed lower values. The AV of the considered resins is rather low and when aiming at polymer with reduced degree of polymerization, statistically some chains with low number of carboxylic groups may be formed. There might even be chains without any hydrophilic moieties which are not soluble at all. For R4, containing AA, both resins were completely turbid after neutralization. AA is more soluble in water than MAA, and forming a polymer with uniform composition can be challenging. Propagation in the water phase is difficult to avoid and an important part of hydrophobic monomers is polymerized in the particles, without anionic moieties to make them amphiphilic [28]. 

**Figure 1:**
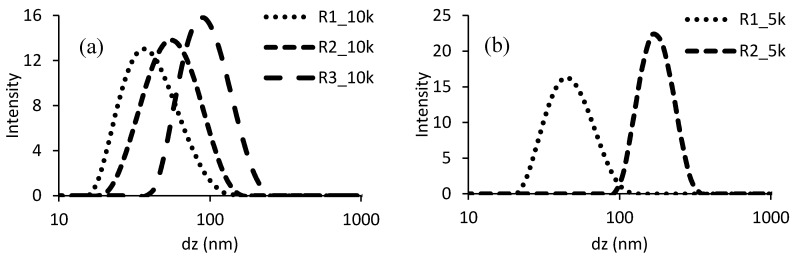
Particle size distributions of neutralized ASRs, Mw equal to 10,000 Da (**a**) or 5000 Da (**b**).

The sizes of aggregates in solution after neutralization (or particles in the case of turbid systems) have also been measured through dynamic light scattering, to understand the colloidal characteristics of the ASRs that will be used as protective colloids to polymerize BA or BMA. Figure 1 presents the particle size distribution of the different resins, comparing ASRs of the same size, since the Mw of the colloid is a crucial point to determine the dissolution behavior when neutralized [29]. The resins R3, size 5000 Da, and both R4 were not sufficiently stable during storage and the data are not available. This last feature is another indication of the inhomogeneity of the mentioned ASRs, composed of hydrophobic chains together with hydrophilic soluble polymer. In addition, an appreciable amount of water soluble polymer can cause colloidal instability, a phenomenon known as “depletion flocculation” [30].

The plots clearly indicate that when focusing on similar degrees of polymerization, introducing EA and replacing MMA provokes an increase on the size of the aggregates/particles (From R1 to R3). The water solubility of these monomers is very similar (≈15 g/L) as well as the molar weight. Therefore, from this point of view no differences should be expected in the emulsion polymerization process of synthesis of the ASRs. The different observed dissolution features and aggregate sizes are probably coming from kinetic considerations involving the reactivity of methacrylates or acrylates in copolymerization with small amounts of S and MAA. These properties are not so easily predictable from literature data, since it is known that the copolymerization constants of acid monomers can vary a lot depending on the solvent and on the ionization degree [31]. Nevertheless, the values of reactivity ratios for systems as closer as possible to that object of this study, as reported in Table 4, confirm that the copolymerization behavior in the two systems should be very different [32,33].

Considering the reported reactivity ratios, MAA should be more uniformly copolymerized with MMA than with EA, therefore more homogeneous and more soluble ASRs are produced employing MMA as main monomer in the formulation, as observed experimentally. 

**Figure 2:**
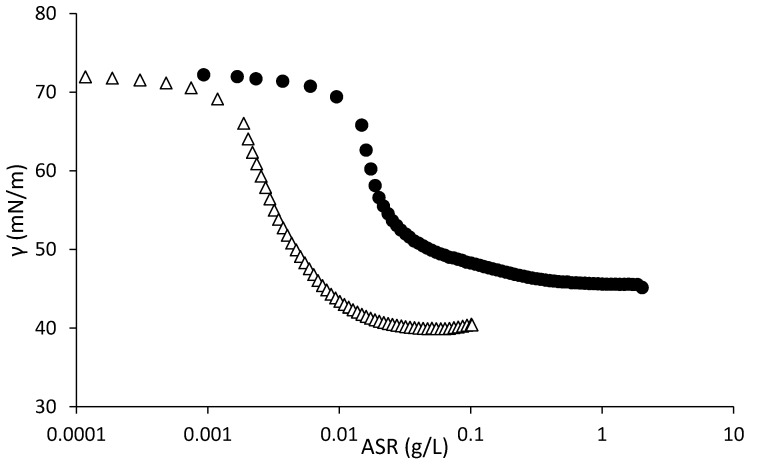
Surface tension as a function of concentration of ASRs at pH equal to 10 for R1 (solid markers) or R3 (empty markers), both of Mw equal to 10,000 Da.

The differences between MMA- or EA-based ASRs were confirmed studying the efficiency of R1 and R3, 10 kDa, in reducing the surface tension of water (Figure 2). The two electrosteric surfactants have different concentration-surface tension profiles, with R3 showing lower surface tension at lower concentration than R1, which is an indication of the higher hydrophilicity of the R1 resin and its more limited adsorption at the air-water interface. As a result, the solubilization ability of R1 aggregates would be lower leading to smaller particles.

### 3.2. Kinetics of Polymerization and Nucleation

**Figure 3:**
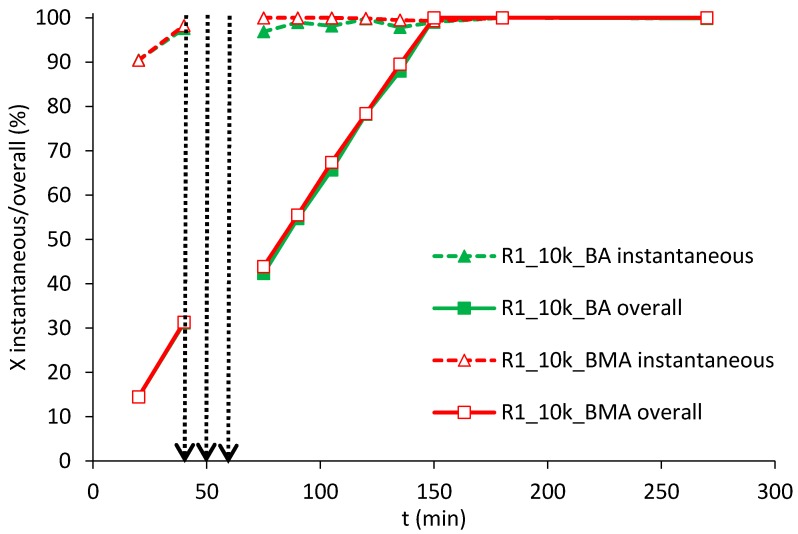
Instantaneous (dashed lines) and overall (continuous lines) conversions using BA (solid markers) or BMA (empty markers), resin R1 of Mw equal to 10,000 Da. The first vertical dashed arrow indicates the end of the first-stage feeding, the second the neutralization step and the third the start of the second-stage feeding.

The considered resins allowed to efficiently produce stable latexes polymerizing both BA and BMA. Kinetics were characterized by starved-feed conditions (i.e., instantaneous conversion close to 100%) with all the resins and both BA or BMA. An example is given in Figure 3 with R1.

**Figure 4:**
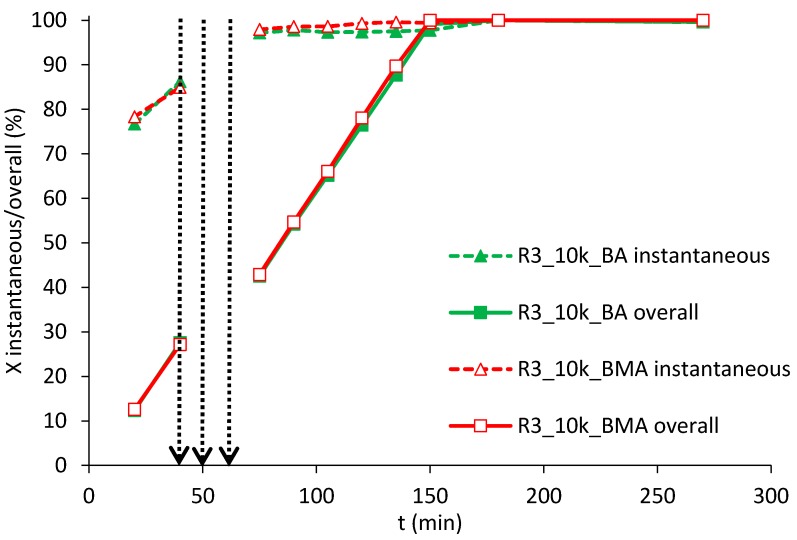
Instantaneous (dashed lines) and overall (continuous lines) conversions using BA (solid markers) or BMA (empty markers), resin R3 of Mw equal to 10,000 Da. The first vertical dashed arrow indicates the end of the first-stage feeding, the second the neutralization step and the third the start of the second-stage feeding.

Remarkably, the same kind of fast kinetics without any retardation has been found using R3 (Figure 4). This particular situation is interesting because it represents somehow the “worst situation”, in terms of hindering of radicals entry, following the description of Peck and Asua [26] and Caballero and coworkers [25]. The resin presents plenty of acrylic moieties and abstractable hydrogens, a charged initiator is used (NaPS) and the high ratio resin:polymer, together with the small size of produced particles, provide huge surface of “hairy layer” which may reduce diffusion of radicals. It has to be pointed out, additionally, that the initiator was used in the amount of 0.5 wt % with respect to the monomer phase; this value is in the common range of normal quantities used in emulsion systems [34] and still lower to those used in the cited works. However, even using these conditions that might provide retardation on the conversion, high instantaneous conversions were found. This feature can be of extreme importance for the safety characteristics of the process, since only small amounts of unreacted monomers are present in the reactor in every moment. Moreover, in the case of feeding of more than one monomer, as commonly happens in industrial processes, compositional drift when polymerizing monomers of different reactivities will be minimized.

**Figure 5:**
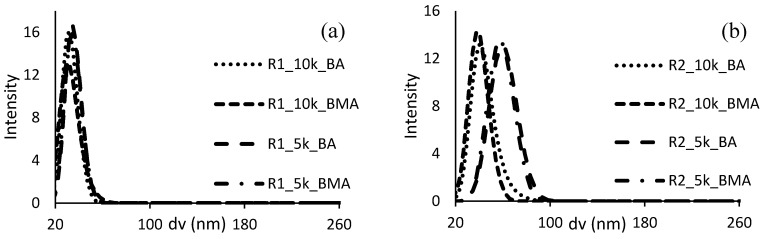

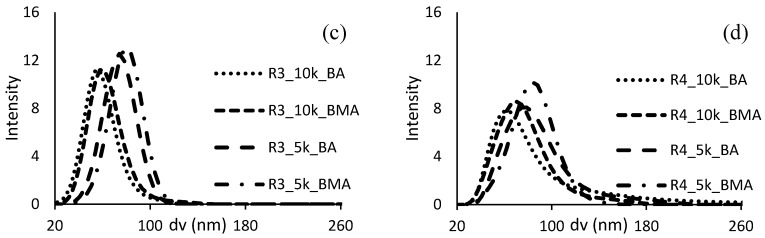
Particle size distributions of final latexes using R1 (**a**), R2 (**b**), R3 (**c**), or R4 (**d**), measured with HDC.

In terms of nucleation, samples of latexes using R1 of molecular weight equal to 10,000 Da were characterized by multimodal distributions by DLS measurements early during the feeding of BA or BMA; it is very likely some large particles are formed in the system as a consequence of coagulative events. Similar observations have been made with R2, with highest molecular weight. These systems start from resins of small size and a lot of surface has to be stabilized, since the number of particles is relevant (around 1 × 10^18^ particles at the beginning of the feeding period). The rest of the latexes, however, presented monomodal distribution and the particle size distributions of the final latexes, as measured by HDC, are presented in Figure 5. In those figures also appear the PSD of the latex from R1 and R2 with Mw 10,000 after filtration through a 1.2 μm filter. 

A difference between the latexes using the same resin with different molecular weights has been observed, especially in resins containing EA (R2, R3 and R4). Moreover, the latexes synthesized with resin R1 present the smaller particle size (coagulated particles were not taken into consideration), which is in concordance with the smallest aggregate/particle size distributions of the neutralized ASRs (Figure 1). Using BA or BMA with the same emulsifier produced latexes with negligible dissimilarity in particle size distribution. Latexes based on R4 (resins formulated with AA) showed rather broad distributions, likely because of the presence of significant amount of water soluble species that cause an increase in viscosity and reduced colloidal stability, also for adsorption of water molecules in the hairy layer of ASR. In the case of R4, the layer of protective colloid is expected to be more hydrophilic, therefore more extended in the water phase and more susceptible of swelling with water.

**Figure 6:**
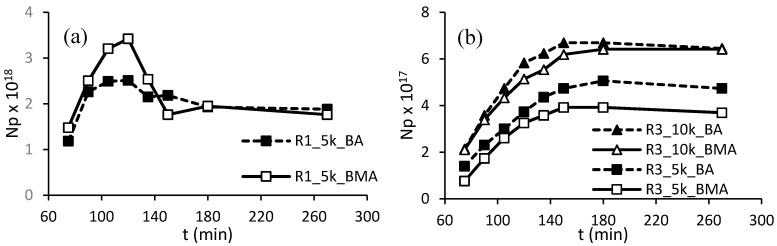
Evolution of number of particles using R1 (**a**) or R3 (**b**).

Focusing on R1 and R3, to point out eventual differences between resins formulated with acrylates or methacrylates, the numbers of particles were calculated in both situations at different time of reaction (Figure 6).

With resin R1 in the molecular weight of 5000 Da, the number of particles is higher than for the latexes synthesized with both R3 resins, because of the smaller aggregate sizes. An increase in the number of particles is evident using R1 with mass of 5000 Da and even clearer employing R3, indicating continuous nucleation during the feeding. Consistent results have been found in analogous works in batch configuration [35,36] or in semibatch systems stabilized with ASRs made with high-temperature processes [27], in which extended nucleation periods have been reported. A possible explanation of the phenomenon could be proposed admitting that electrosteric surfactants are quite oil-soluble and can partition between the water and the organic phase; this implies that there may be a continuous release of emulsifier molecules from the organic phase as long as more polymeric chains from the water phase are adsorbed onto the growing particles [37]. With the same resin, slightly higher nucleation can be recognized using BA in respect to BMA. BA is a bit more hydrophilic than BMA, therefore the parking area of the same surfactant onto BA is higher than onto BMA, and bigger particles are formed in the latter case.

### 3.3. Microstructure Features

**Figure 7:**
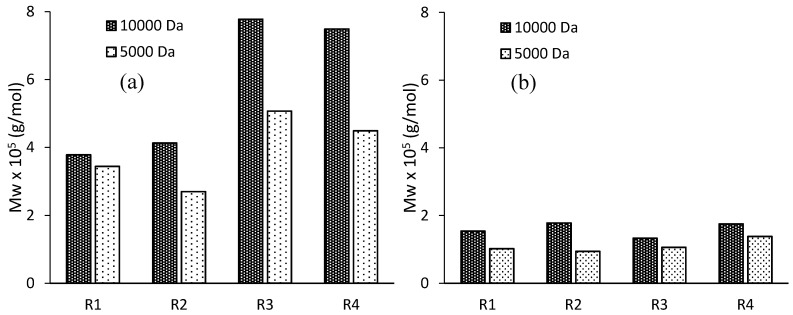
Weight-average molecular weights of the synthesized latexes in the polymerization of BA (**a**) or BMA (**b**).

The measured average molecular weights of the synthesized latexes are presented in Figure 7.

Considering the same resin, latexes polymerized with BA always had a higher molecular weight than the BMA case. This observation can be explained knowing that the kp of BA is roughly one order of magnitude higher than that of BMA [38,39], as well as taking into account that BA termination is mostly by combination while for BMA is by disproportionation.

Comparing ASRs with the same composition but of different molecular weights, and both used to stabilize the same monomer, the latexes containing the smaller resin are always smaller in average molecular weight. The size of particles is bigger in the case of the resins with molecular weight equal to 5000 Da, so the frequency of radical entry is higher yielding to lower molecular weight. Focusing on latexes based on resins of the same size, there is not a clear trend. The most significant variation is with the polymerization of BA synthesized with resins R3 and R4, in which a clear increment on molecular weight is observed. In this case, differences on particle sizes hardly can justify the observed results. Resins R3 and R4 are the ones with the higher amount of sites for abstraction (75 wt % of EA in R3, 75 wt % of EA and 15 wt % of AA in R4). Therefore, grafting reactions based on abstraction pathways may occur because of the presence of acrylic moieties in the resins causing branched structures after the incorporation of the electrosteric stabilizer in growing chains. These branched structures are characterized by an increased hydrodynamic radius and are eluted earlier in the GPC chromatogram. These evidences seem to indicate qualitatively that resins with more sites for abstractions effectively allow an increasing of grafting between the ASR and the polymeric particles. 

Therefore, in order to check the possible effect of the ASR composition on microstructural features, the grafting between the electrosteric surfactant and the particles was evaluated. 

Indeed, grafting may occur by hydrogen abstraction, followed by propagation of the new-formed radical on the resin. The abstraction reaction has a higher probability to occur when acrylic moieties are present, because the resulting tertiary radical in α position with respect to the carbonyl group can be stabilized by resonance with same group. Because of that, grafting through hydrogen abstraction is expected to be relevant when acrylic acid and/or acrylic esters are present on the structure of the ASR [25]. 

**Figure 8:**
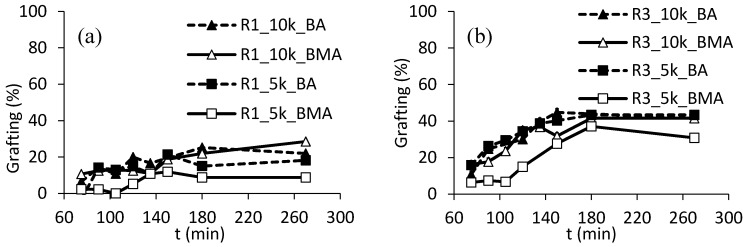
Grafting amounts using R1 (**a**) or R3 (**b**).

Figure 8 shows the development of the grafting of resins R1 and R3 during feeding. As expected, higher levels of linking are observed for resin R3, that is, when acrylates moieties are present on the backbone of the resin. While there is not any appreciable difference among syntheses with longer ASRs and the shorter one using BA, the syntheses using the ASRs with Mw equal to 5000 Da to polymerize BMA are in both cases characterized by slightly lower grafting amounts in the final latexes. A possible explanation is that in order to graft significant amounts of smaller ASRs, more grafting events are required, and normally it is easier to obtain high levels of grafting (on a mass basis) with longer chains. In addition, BA is more efficient in hydrogen abstraction than BMA, since radicals of BA are of secondary type, whereas those of BMA are tertiary, resulting in higher degree of grafting.

**Figure 9:**
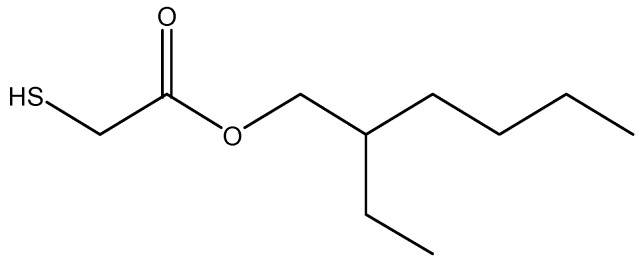
Chemical structure of 2-Ethylhexyl thioglycolate (EHTG).

Considering the latexes based on R1, the analysis revealed that some grafting can be obtained in this system (less than 30% in the final latexes). This is rather surprising, since there are no acrylic esters or acrylic acid in the used formulation. However, EHTG, which has been used as CTA, has an acrylic structure with two atoms of hydrogen in α position with respect to the carbonyl group (Figure 9). The presence of a sulfur atom on the same carbon atom might also increase the propensity towards abstraction. Thus, abstraction and grafting can likely start from this moiety, which is sufficiently accessible because of its terminal position on the polymeric chains. 

**Figure 10:**
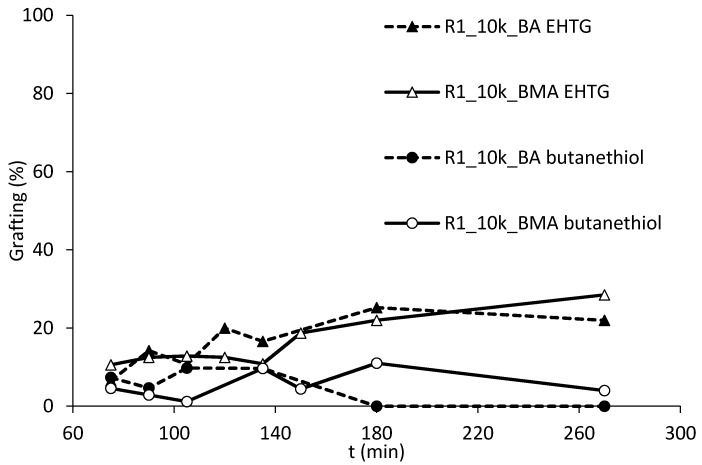
Comparison of amounts of grafting using R1, Mw equal to 10,000 Da, employing EHTG or 1-butanethiol as CTA.

To test this hypothesis, R1 was produced with the same experimental conditions replacing EHTG with 1-butanethiol targeting for a molecular weight of 10,000 Da. The syntheses proceed with similar kinetics and similar nucleation behavior. Grafting amounts in the successive polymerizations of BA or BMA are presented in Figure 10.

The profiles using 1-butanethiol as CTA instead of EHTG confirm that negligible amounts of grafting are obtainable when the CTA does not have abstractable hydrogens. The use of a suitable CTA can therefore allow introducing grafting points in the structure of the electrosteric stabilizer, even if resins without acrylates are desired, e.g., when hard resins are required. 

As an overall, grafting levels cannot reach very high extents, not even when the resin is specifically formulated to enhance hydrogen abstraction (R3). This finding is an agreement with previous data indicating that in systems where grafting can proceed through hydrogen abstraction and incorporation of resin through reactive double bonds; this latter pathway is the predominant one and responsible for very high chemical linkage [27].

## 4. Conclusions

Electrosterically stabilized latexes made with a one-pot strategy have been synthesized and characterized as concerns kinetics features, nucleation behavior and microstructural features including molecular weight and grafting between the protective colloid and the particles. The strategy is of particular interest because it allows the synthesis of the electrosteric emulsifier and the dispersion in the same reactor, without the use of organic solvents. The studied resin presented AVs which were lower than those of commercial ASRs, however good stability of the resulting latexes was achieved.

All resins were produced with high final conversions. The dissolution behavior depended on the molecular weight and the composition of the resins. In particular, resins characterized by uniform distribution of carboxylic groups showed clearer dispersions upon neutralization, with lower sizes of the aggregates. The difference in reactivity ratios of the copolymerized monomers is the main parameter affecting the monomer sequence distribution of the resin. The ASRs were subsequently used in-situ after neutralization to stabilize BA or BMA. Fast conversions were observed in all the experimental conditions, differently to what has been claimed in the literature with analogous systems. This feature could provide valuable benefits from the industrial point of view. The stage of nucleation generally extended over the entire feeding time. Grafting was measured and it was found to be higher using resins with acrylate esters in the formulation, since hydrogen abstraction in α position of the carbonyl functionality and propagation of the formed tertiary radicals is the only possible reactive pathway in these specific systems. As an overall, grafting levels are nevertheless moderate even in systems designed to enhance abstraction.

## Figures and Tables

**Table 1:** Formulations used for the syntheses of the ASRs.

RESIN	MMA (% *w*/*w*)	S (% *w*/*w*)	EA (% *w*/*w*)	MAA (% *w*/*w*)	AA (% *w*/*w*)	AV (mg KOH/ g Resin)
R1	75	10	-	15	-	98
R2	35	10	40	15	-	98
R3	-	10	75	15	-	98
R4	-	10	75	-	15	117

**Table 2:** Standard formulation used for the emulsion polymerizations.

		Amount (g)
**Initial charge**	Water	255.00
	SDS 15%	4.00
**Initiator solution**	Water	19.92
	NaPS	1.50
**First stage pre-emulsion**	Water	60.00
	SDS 15%	3.40
	Monomers	90.00
	EHTG	3.30
**Base**	Ammonia 25%	10.74
**Second stage pre-emulsion**	Water	111.00
	SDS 15%	4.60
	Monomer	210.00

**Table 3:** Weight-average molecular weight (Mw) and transmittance of neutralized ASRs.

Resin	Mw (g/mol)	Transmittance (%)	Resin	Mw (g/mol)	Transmittance (%)
R1_10k	9700	84 ± 1	R1_5k	4600	48 ± 3
R2_10k	10,600	91 ± 1	R2_5k	5100	0
R3_10k	11,700	72 ± 3	R3_5k	5300	2 ± 1
R4_10k	11,100	0	R4_5k	6000	0

**Table 4:** Reactivity ratios for copolymerization of MAA with EA or MMA.

RESIN		
	rMMA	rMAA
**R1**	0.6	0.3
	rEA	rMAA
**R3**	0.2	2.9
